# What's Wrong with This Picture? A Critical Review of Current Centers for Medicare & Medicaid Services Coverage Criteria for Continuous Glucose Monitoring

**DOI:** 10.1089/dia.2021.0107

**Published:** 2021-09-01

**Authors:** Rodolfo J. Galindo, Christopher G. Parkin, Grazia Aleppo, Anders L. Carlson, Davida F. Kruger, Carol J. Levy, Guillermo E. Umpierrez, Janet B. McGill

**Affiliations:** ^1^ Emory University School of Medicine, Atlanta, Georgia, USA.; ^2^ Center for Diabetes Metabolism Research Emory University Hospital Midtown, Atlanta, Georgia, USA.; ^3^ Hospital Diabetes Taskforce, Emory Healthcare System, Atlanta, Georgia, USA.; ^4^ Clinical Research, CGParkin Communications, Inc., Henderson, Nevada, USA.; ^5^ Division of Endocrinology, Metabolism and Molecular Medicine, Northwestern University, Chicago, Illinois, USA.; ^6^ International Diabetes Center, Minneapolis, Minnesota, USA.; ^7^ Regions Hospital & HealthPartners Clinics, St Paul, Minnesota, USA.; ^8^ Diabetes Education Programs, HealthPartners and Stillwater Medical Group, Stillwater, Minnesota, USA.; ^9^ University of Minnesota Medical School, Minneapolis, Minnesota, USA.; ^10^ Division of Endocrinology, Diabetes, Bone & Mineral, Henry Ford Health System, Detroit, Michigan, USA.; ^11^ Division of Endocrinology, Diabetes, and Metabolism, Icahn School of Medicine at Mount Sinai, New York City, New York, USA.; ^12^ Mount Sinai Diabetes Center and T1D Clinical Research, Icahn School of Medicine at Mount Sinai, New York City, New York, USA.; ^13^ Division of Endocrinology, Metabolism, Emory University School of Medicine, Atlanta, Georgia, USA.; ^14^ Diabetes and Endocrinology, Grady Memorial Hospital, Atlanta, Georgia, USA.; ^15^ Division of Endocrinology, Metabolism and Lipid Research, School of Medicine, Washington University in St. Louis, St. Louis, Missouri, USA.

**Keywords:** Continuous glucose monitoring, Centers for Medicare & Medicaid Services, Insurance coverage, Type 1 diabetes, Type 2 diabetes

## Abstract

Numerous studies have demonstrated the clinical value of continuous glucose monitoring (CGM) in type 1 diabetes and type 2 diabetes populations. However, the eligibility criteria for CGM coverage required by the Centers for Medicare & Medicaid Services (CMS) ignore conclusive evidence that supports CGM use in various diabetes populations that are currently deemed ineligible. This article discusses the limitations and inconsistencies of the CMS eligibility criteria relative to current scientific evidence and proposes workable solutions to address this issue and improve the safety and care of all individuals with diabetes.

## Introduction

Among individuals ≥65 years, the prevalence of diabetes has now reached over 30%.^1–4^ In the recent Centers for Disease Control & Prevention 2020 report, it was found that an additional 7.3 million adults who met laboratory criteria for diabetes were not aware of their condition.^1^

The increasing prevalence in the United States continues to be a significant and growing health issue. However, the concern is greatest among racial and ethnic minority populations, in which the prevalence of diabetes and its debilitating complications is significantly higher than the broader white population.^5^ According to the latest estimates, the prevalence of diagnosed diabetes is highest within the Native American (14.7%), Hispanic American (12.5%), and non-Hispanic black American (11.7%) populations compared with non-Hispanic white (7.5%) populations.^1,5^

**FIG. 1.**
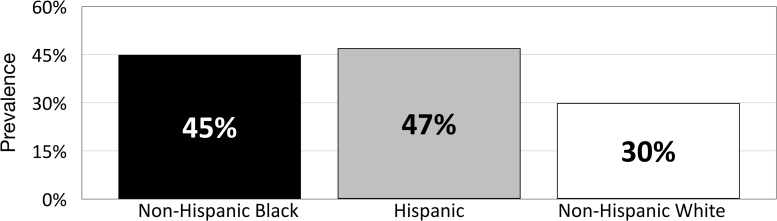
Diabetes prevalence within the Medicare population by race/ethnicity.^3^

These disparities are most notable among the Medicare diabetes population. Key findings from a 2017 report from Centers for Medicare & Medicaid Services (CMS) revealed that diabetes prevalence was higher among non-Hispanic black (30.0%) and Hispanic beneficiaries (26.7%) compared with non-Hispanic white beneficiaries (18.0%), but that significantly lower percentages of black (65.2%) and Hispanic (64.3%) than white (79.4%) beneficiaries are aware that Medicare helps pay for diabetes testing supplies and education.^6^ A 2018 survey by the Kaiser Family Foundation reported that the prevalence of diabetes was notably higher within non-Hispanic black and Hispanic Medicare populations compared with the non-Hispanic white population (Fig. 1).^3^

It is important to note that these statistics are from government agency reports, which include comprehensive descriptions of the various scientific methodologies used to generate the data. However, there appears to be disconnection between how science is used to assess a problem but not utilized in solving problems.

Although the medical community traditionally relies on high-quality scientific evidence when developing clinical guidelines for managing diabetes and other conditions, many regulatory agencies and public and private insurers tend to ignore the evidence and take a different path when establishing coverage eligibility criteria for medications and medical devices. This is particularly apparent in the current eligibility criteria for use of personal continuous glucose monitoring (CGM), which deny millions of Americans access to this proven technology.

This article discusses the limitations and inconsistencies of the CMS eligibility criteria relative to current scientific evidence and proposes workable solutions to address this issue and improve the safety and care of all individuals with diabetes.

## Evidence Supporting CGM Use in Various Diabetes Populations

### Type 1 diabetes

The clinical efficacy of CGM has been demonstrated for over a decade in numerous studies of individuals with type 1 diabetes (T1D) regardless of insulin delivery method.^7–23^ Benefits of CGM use in this population include reductions in HbA1c,^7,9,11,17,18,24–28^ fewer severe hypoglycemia events,^25,26,29^ increased time within target glucose range (TIR),^11,18,19,30^ and reductions in time below range.^11,18^ Large observational registry and database studies have also shown an association between CGM use and significant reductions in hospitalizations for severe hypoglycemia and diabetic ketoacidosis (DKA)^25,26,29,31^ For example, in the RESCUE trial, a multicenter prospective observational cohort study of T1D adults (*n* = 515) treated with insulin pump therapy, switching from self-monitoring of blood glucose (SMBG) to CGM during the 12-month observation period, was associated with significant reductions in the number of patients hospitalized for severe hypoglycemia, a decrease of 73% (from 11.9% to 3.2%),^29^ as well as DKA-related hospitalizations decreased by 80% (from 4.6% to 1.1%).

### Problematic hypoglycemia regardless of treatment regimen

Problematic hypoglycemia has been well-documented in individuals with T1D and type 2 diabetes (T2D) who are treated with intensive insulin therapy, and recent studies have also reported problematic hypoglycemia in T2D patients who are treated with less intensive insulin regimens or no insulin.^32–34^ This is particularly concerning among older patients, who are at significantly higher risk for severe hypoglycemia compared with younger patients due to their age, diabetes duration, insulin therapy duration, glucose variability, and higher prevalence of impaired hypoglycemia awareness.^35–40^ Early and recent studies, utilizing CGM documentation, have demonstrated an increased risk of severe hypoglycemia among patients ≥65 years treated with less intensive insulin regimens or oral antidiabetic medications.^16,41,42^ Importantly, as reported by Weinstock et al., the risk of severe hypoglycemia is not associated with HbA1c or mean glucose measured by SMBG.^35^ Recent studies have shown that use of CGM compared with SMBG significantly reduces glycemic variability,^17^ a risk factor for severe hypoglycemia,^43–45^ and the time spent in hypoglycemia^21,22^ among T2D adults treated with intensive insulin therapy.

In a recent study by Pratley et al., 203 older adults (≥60 years) were randomized to CGM or SMBG use.^46^ At 6 months, CGM use was associated with decreases in severe hypoglycemia compared with SMBG, showing significant reductions in severe hypoglycemia incidence rates (per 100 person-years) compared with SMBG (1.9 vs. 22.4, respectively, *P* = 0.02). CGM use was also associated with reductions in the percentage of time spent <70 mg/dL (from 5.1% to 2.7%) versus increases with SMBG use (from 4.7% to 4.9%), *P* < 0.001. Considering that the average cost for a hypoglycemia-related hospitalization among Medicare beneficiaries is estimated at >$10,000,^47^ the 10-fold decrease in severe hypoglycemia incidence rates reported by Pratley is notable. It is therefore reasonable to suggest that CGM use would significantly reduce health care costs while improving the safety of T2D patients treated with less intensive insulin regimens, particularly in older patients with frequent severe hypoglycemia or impaired hypoglycemia awareness.

Apart from the acute clinical outcomes resulting from severe hypoglycemia events, these episodes also impact patients' willingness to adhere to their prescribed therapy, which can result in suboptimal glycemic control and increased risk of long-term complications.^48,49^ An international survey of 27,585 diabetes patients found that 25.8% to 46.7% of people with T2D reduced their insulin dosages in response to hypoglycemia.^50^ CGM use has been shown to reduce hypoglycemia fear and increase patient confidence in avoiding/treating hypoglycemia.^7,48^ This is particularly relevant in patients with problematic hypoglycemia.

### Pregnancy

The CONCEPTT trial assessed the clinical impact of CGM use versus SMBG within a cohort of 325 women with T1D who were pregnant (≤13 weeks gestation) or planning to become pregnant.^51^ Significant increases in time in target range with CGM compared with SMBG use (68% vs. 61%; *P* = 0 · 0034, respectively) were observed. CGM users also experienced improved fetal outcomes, including lower incidence of large for gestational age (*P* = 0.0210), fewer neonatal intensive care admissions lasting more than 24 h (*P* = 0.0157), fewer incidences of neonatal hypoglycemia (*P* = 0 · 0250), and shorter length of hospital stay (*P* = 0 · 0091).

### Chronic kidney disease

Although few studies of CGM use in patients with advanced chronic kidney disease (CKD) have been conducted, Joubert et al. demonstrated a strong association between iterative CGM and frequent treatment changes and improved glycemic control without increased risk of hypoglycemia in diabetes patients on chronic dialysis.^52^ The value of CGM has also been shown in monitoring and managing glycemic levels in nondiabetic patients with end-stage renal disease who are undergoing dialysis.^53^ A recent analysis of the T1D Exchange registry data set found that fewer participants using CGM experienced an adverse renal outcome compared with those with no history of CGM use.^54^ An added benefit of CGM use in this population is that it provides additional data regarding glycemic status via the Glucose Management Indicator, which is a more reliable method of monitoring long-term glycemic control compared to HbA1c.^55^

### Telemedicine

Numerous meta-analyses and systematic reviews of randomized controlled trials have demonstrated that the addition of telemedicine and telemonitoring interventions in patients with T1D and T2D results in reductions in HbA1c,^56–61^ incidence of severe hypoglycemic events,^60^ diabetes-related distress,^62^ and improvements in medication adherence.^63^ A 2020 systematic review and meta-analysis of remote monitoring and telehealth technologies in patients with diabetes reported significant reductions in HbA1c levels.^57^ Importantly, a subgroup analysis showed that remote patient monitoring is effective for patients who are residents of cities, especially when using monitoring software (e.g., Dexcom Clarity, Abbott LibreView, Medtronic CareLink) as a component of the intervention.

Use of digital diabetes technologies that transmit and present CGM data in a standardized report, such as the Ambulatory Glucose Profile (AGP), support analysis of patient glucose data to inform treatment decisions. When shared with the patient, the AGP results were found to be an effective basis for education, helping achieve better understanding of glycemic variability and increasing involvement in diabetes self-management.^64^ Most recently, use of remotely monitored CGM data as a component of a comprehensive telemedicine program showed statistically significant HbA1c reductions (*P* < 0.001) in a cohort to 594 T2D adults treated with less intensive insulin therapy or noninsulin medications.^15^

## Current CMS Eligibility Criteria for CGM Coverage

On January 12, 2017, CMS initiated coverage for use of CGM among insulin-treated diabetes beneficiaries who met the following eligibility criteria: (1) diagnosis of diabetes; (2) documentation of frequent SMBG (defined as testing ≥4 times daily); (3) treatment with intensive insulin therapy (defined as ≥3 insulin injections per day or use of a Medicare-covered insulin pump); (4) frequent adjustment of insulin dosages based on blood glucose measurements; (5) face-to-face consultation with clinician before initiating CGM; and (6) follow-up face-to-face clinical consultations every 6 months. However, despite the demonstrated clinical benefits of CGM, many Medicare beneficiaries with diagnosed diabetes do not meet these eligibility criteria and are thus denied access to CGM technology.^65^ Most clinicians who care for persons with diabetes are unfamiliar with the criteria and documentation required to obtain a CGM for eligible patients, further limiting use by patients treated in primary care and clinic settings.

## Recommended Changes to Current Eligibility Criteria

### Eliminate SMBG frequency requirement

Requiring SMBG frequency of ≥4 times daily is not only overly restrictive but also medically unfounded and should not be included in Medicare coverage criteria. In the Ruedy study, 52% of the CGM users reported SMBG frequency of <4 tests per day at baseline, with no association between HbA1c reductions and baseline SMBG frequency.^17^ A similar absence of association between previous SMBG frequency and positive clinical outcomes with CGM use has been observed in other large, randomized trials.^10,21,31^

In the DIAMOND T2D study, the mean self-reported SMBG frequency for the CGM and SMBG groups was 3.3 and 3.2, respectively, at baseline.^10^ At 6 months, the mean change in HbA1c was significantly greater in the CGM group (−1.0) compared with SMBG users (−0.6%), *P* = 0.005. No association between baseline SMBG frequency and CGM outcomes was observed. Similarly, post hoc analysis of data from the REPLACE study showed no association between prior SMBG frequency (<4 × vs. ≥4 × daily) and outcomes.^21^

Findings from a recent retrospective claims that data analyses have also shown no association between prior SMBG frequency and reductions in acute diabetes events (ADE) associated with CGM use.^31^ In the analysis, a cohort of 12,521 individuals with T1D and T2D experienced reductions in ADE from 0.245 to 0.132 events/patient-year (*P* < 0.001), with similar reductions observed in patients testing <4 and ≥4 times per day.^31^ Importantly, many Medicare beneficiaries are unable to meet the ≥4 times per day fingerstick testing requirement due to limited dexterity or restrictions on the number of strips allowed by Medicare.^66^ Although ≥4 times per day fingerstick testing is required for coverage, Medicare only covers 100 test strips per month (∼3 strips/day) unless clinicians are willing to provide additional documentation supporting more frequent testing.

### Eliminate intensive insulin regimen requirements for T2D

Studies have demonstrated that use of CGM by T2D patients confers significant reductions in HbA1c levels,^10,13–15,17,24,67,68^ significant increases in percent time in range (defined as glucose values between 70 and 180 mg/dL, %TIR),^10,17^ significant decreases in percent time below range (defined as glucose values <70 mg/dL, %TBR),^21,22^ and significant reductions in diabetes-related hospitalizations^31,69^ regardless of insulin regimen. Although a substantial number of T2D Medicare beneficiaries are treated with less-intensive insulin regimens, they are at much higher risk for severe hypoglycemia than younger patients.^16,35,36,42^

### Eliminate requirement for frequent insulin dosage adjustments based on glucose values

The requirement for a documented history of frequent insulin dosage adjustment based on SMBG values is unrealistic and burdensome for both health care providers and patients, and there is no evidence demonstrating its value as a predictor of successful CGM use. Moreover, this requirement ignores the safety features of CGM, which include the automated alarms and alerts that warn patients of current or impending hypoglycemia/hyperglycemia, enabling them to take immediate remedial action. Importantly, several instances of U-500- and premixed insulin-related errors have been reported, resulting in severe hypoglycemia.^70^ These insulin preparations are generally administered only once or twice daily, which means that patients using these medications are not currently eligible for CGM use, denying them an important safety device. Finally, the specific wording of the requirement for “injecting” insulin fails to address other options for insulin administration (e.g., insulin infusion using a pump and inhaled insulin).

### Include telemedicine as an option for clinical consultations

Apart from the fact that FDA labeling for current CGM systems does not require in-person training, numerous studies have shown that use of telemedicine consults confers significant glycemic^56–61^ and psychosocial^62,63^ benefits. Moreover, the successful utilization of telemedicine consults during the COVID-19 pandemic^71–75^ highlights the clinical value and utility of this approach to health care delivery. It is hoped that this temporary allowance by CMS will continue after the pandemic ends, and that Congress will include all patients who choose this option, not just those in rural communities. Because many older patients may not have access or unable to use more advanced telemedicine technologies, remote consultations via telephone must be an option for meeting the 6-month consult requirement for verifying continued CGM use.

### Streamlined and standardized documentation requirements for obtaining coverage

Because obtaining CMS coverage for CGM places an unwarranted burden on clinicians and office staff who must gather and submit substantial documentation,^76^ many clinicians are unwilling or unable to meet the documentation requirements. This, in turn, can be detrimental to patient care. In a 2017 survey by the American Medical Association, 92% of the 1000 physicians surveyed reported that prior authorizations delay patient treatment and negatively impact clinical outcomes.^77^ Importantly, 78% of respondents reported that the documentation requirements associated with obtaining preauthorizations sometimes (57%), often (19%), or always (2%) result in their patients discontinuing their prescribed therapy. This is evidenced by the apparent racial/ethnic disparities relative to CGM eligibility within the Medicare population. A recent analysis of 2018 Medicare data found that the majority (69%) of insulin-treated beneficiaries do not qualify for CGM coverage under the current ≥4x daily blood glucose testing requirement.^65^ However, the percentage of ineligible non-Hispanic black (≥74%) and Hispanic (≥75%) beneficiaries is notably higher than observed in non-Hispanic white (68%) beneficiaries.

### Provide clear guidance to Durable Medical Equipment suppliers

Beneficiary access to CGM is further hindered by CMSs lack of clarity in providing guidance to Durable Medical Equipment (DME) suppliers for determining the medical necessity for CGM in many of the coverage claims they receive. As a result, claims are often rejected by suppliers to avoid potential financial penalties that could be imposed by CMS.

## Proposed Eligibility Criteria for CGM Coverage

To expand access to all individuals who would benefit from CGM, streamline clinician documentation requirements and clarify DME supplier guidance, the panel recommends that CMS modify its eligibility requirements to include all Medicare beneficiaries who meet any one of the first four criteria below, and who also meet the fifth criterion:

1. Diagnosed with T1D.
2. Diagnosed with T2D and treated with any insulin regimen.
3. Diagnosed with T2D and documented problematic hypoglycemia regardless of diabetes therapy. This would include a history of at least one of the following conditions: Level 2 (moderate) hypoglycemia, characterized by glucose levels ≤54 mg/dL; Level 3 (severe) hypoglycemia, characterized by physical/mental dysfunction requiring third-party assistance; or nocturnal hypoglycemia
4. Advanced CKD at risk for hypoglycemia.
5. In-person or telemedicine consultation with the prescribing health care provider before CGM initiation and every 6 months thereafter while continuing CGM therapy. (Coverage for telemedicine consults should be available for all patients regardless of geographic location.)

Table 1 presents the link between these proposed criteria and their supporting evidence.

**Table 1.** Proposed Eligibility Criteria and Supporting Evidence

Criterion	Supporting evidence
1. Diagnosed with T1D.	CGM use confers: Significant reductions in HbA1c.^7,9,11,17,18,24–28^ Significant reductions in severe hypoglycemia events.^25,26,29^ Significant increases in %TIR.^11,18,19,30^ Significant decreases in %TBR.^11,18^ Significant reductions in diabetes-related hospitalizations.^25,26,29,31^ Significant improvements in treatment satisfaction with less diabetes distress^25,27,^^78^
2. Diagnosed with T2D and treated with any insulin therapy.	CGM use confers: Significant reductions in HbA1c.^10,13–15,17,24,67,68,79^ Significant increases in %TIR.^10,17^ Significant decreases in %TBR.^21,22^ Significant reductions in diabetes-related hospitalizations.^31,69^
3. Diagnosed with T2D and documented problematic hypoglycemia regardless of diabetes therapy. This would include a history of at least one of the following conditions: Level 2 (moderate) hypoglycemia, characterized by glucose levels ≤54 mg/dL. Level 3 (severe) hypoglycemia—characterized by physical/mental dysfunction requiring third-party assistance. Nocturnal hypoglycemia.	Older diabetes patients are at increased hypoglycemia risk: T2D patients treated with antihyperglycemic medications (e.g., insulin, sulfonylureas) are at higher risk for hypoglycemia than those treated with nonhypoglycemia medications (e.g., metformin).^16^ T2D patients ≥65 years treated with basal insulin (typically one injection per day) are at increased risk for severe hypoglycemia.^42^ A key driver of hypoglycemia risk is impaired hypoglycemia awareness.^35,70^ CGM use confers: Significant reductions in diabetes-related hospitalizations, including severe hypoglycemia events.^31,69^ Significant reductions in hypoglycemia fear and increases patient confidence in avoiding/treating hypoglycemia,^7,80^ thereby supporting treatment adherence.^48,49^
4. CKD.	CGM use facilitates: More frequent treatment changes and improved glycemic control without increased risk of hypoglycemia.^52^ Effective monitoring and managing glycemic levels in non-diabetic patients with ESRD undergoing dialysis.^53^
5. In-person or telemedicine consultation with the prescribing health care provider before CGM initiation and every 6 months thereafter while continuing CGM therapy.	Use of telemedicine consults: Significantly reduces HbA1c.^56–61^ Reduces the incidence of severe hypoglycemic events.^60^ Significantly reduces diabetes-related distress.^62^ Significantly improves medication adherence.^63^ Effectively addresses the obstacles caused by the COVID-19 pandemic.^71–75^ Are more effective for patients who are residents of cities and using the websites as their intervention method.^57^ Use of downloaded CGM data into standardized reports: Supports patient education.^64^ Enhances patient engagement in their self-management.^64^

%TIR = percentage of time in glucose range (70–180 mg/dL); %TBR = percentage of time below target ranges (<70 mg/dL, <54 mg/dL).

CGM, continuous glucose monitoring; CKD, chronic kidney disease; ESRD, end-stage renal disease; T1D, type 1 diabetes; T2D, type 2 diabetes.

## Conclusion

A substantial and growing body of evidence clearly demonstrates the clinical benefits of CGM in individuals with T1D and T2D regardless of their current therapy and prior glucose monitoring frequency.^21,31,67–69,79,81,82^ The medically unfounded Medicare eligibility criteria for CGM coverage and lack of clear guidance to DME suppliers deny access to CGM among a substantial population of Medicare beneficiaries with diagnosed diabetes. To the extent that Medicare's coverage criteria are adopted by Medicaid or commercial payers, these policies have a negative ripple effect on access to CGM.

Restricted access to CGM is particularly concerning within populations of patients with racial and ethnic diversity. As reported by Taylor et al. in an early study of racial and ethnic disparities in diabetes care, non-Hispanic black patients had a 25% lower odds ratio for achieving HbA1c levels of <8.0% and 58% higher odds ratio of sustaining HbA1c levels at >9.0%.^83^ An analysis of unpublished Medicare data revealed that a notably higher percentage of non-Hispanic black (>75%) and Hispanic (>75%) insulin-treated beneficiaries are ineligible for CGM based on their current SMBG frequency. Importantly, although an early study by Groeneveld et al. found that there were inconsistent differences between non-Hispanic black and non-Hispanic white patients in their openness to using innovative technology,^84^ a recent study of 300 young adult T1D patients found notably lower use of CGM among non-Hispanic black (28%) and Hispanic (37%) patients compared with non-Hispanic white (71%) patients.^85^

Many researchers add a qualifier at the end of their published study reports, explaining how additional studies are needed to further understand their findings and/or support their conclusions. For this report, no such qualifier is needed. Given the demonstrated clinical benefits and lower rates of diabetes-related hospitalizations associated with CGM use, limiting access to CGM technology achieves neither cost-efficiencies nor clinical efficacies. We believe our evidence-based recommendations for modifying current eligibility criteria both streamline the administrative processes for documenting medical necessity, expand access to CGM and improve the safety of our most vulnerable diabetes population.
